# Cases of Abcess Connected with the Urinary Bladder

**Published:** 1846-02

**Authors:** 


*Cases of Abcess connected with the Urinary Bladder. Communicated by* Dr. Spittal, Edinburgh.—Case 1. The following imperfect notes of this case may be interesting on account of the rarity of the attendant circumstances. The patient, J. W., a man aged thirty, while engaged in sinking a pit, was struck on the back by the falling in of a stone upon him. From this period, he lost all power of motion and sensation in the lower extremities; and could not retain his excretions. For about eight years he continued much in the same condition, the urine flowing incontinently; and he neglected the use of the catheter. His general health during this time remained good; but his lower extremities became very much emaciated, and, owing to his complete want of sensation, the extremities of his toes sometimes became a prey to mice, which left sanguinary evidence of their partiality. In the month of March, of the eighth year after the injury, after considerable uneasiness in the region of the bladder, an abscess formed in the hypogastrium, midway between the pubes and umbilicus, in the mesial line. This abscess gave way externally, as well as into the bladder, and along with pus, *all the urine flowed for seven weeks from this opening in the parietes of the abdomen*, the use of the catheter having been objected to. About the end of the above period, the urine flowed from the urethra as before, his general health having suffered very little during the continuance of the circumstances related. He lived for more than a year afterwards, and died rather suddenly, after suffering from acute pain in the head, followed by convulsions, delirium and coma.

*Examination of the body.*—The spine projected, in a somewhat conical form, about the lowest dorsal vertebræ. The spinal cavity was found much diminished in its calibre, and the spinal cord, having been much pressed upon, was considerably atrophied at this point. The urinary bladder was enlarged and much thickened in its coats, and presented at its fundus a cicatrix corresponding to an external cicatrix, in the site of the abscess, on the surface of the abdomen, where the bladder had formed a firm attachment to the parietes. The cicatrices showed that the perforation, by the abscess, had occurred in the upper part of that portion of the bladder uncovered by the peritoneum. The ureters were irregularly thickened and enlarged; and the pelvis of both kidneys was enlarged, while the substance of these organs had become atrophied, but seemed otherwise healthy. The condition of the bladder, ureters, and kidneys described, probably resulted from the mechanical pressure of the urine on these parts; for that fluid would only find its way by the urethra, after full distension of the bladder, when most likely the valvular entrance of the ureters was rendered of no avail. This state of matters may also account, perhaps, for the occurrence of the abscess itself; and the cause of death may have been intimately connected with the diminished eliminating powers of the kidneys in their atrophied condition, while, at the same time, labouring under a mechanically diminished supply of blood,—the most probable cause of the condition described.

On mentioning the features of this case to Mr. Goodsir, that distinguished anatomist informed me of a very interesting and somewhat similar case, of which he was good enough to send me the notes which I subjoin.

Case 2. T. M., aged seventy, a carpenter, of robust constitution, had, from an early period of his life, been liable to retention of urine, after indulging in the use even of the smallest quantity of ardent spirits. These attacks of retention were invariably, and easily, relieved by a single introduction of the catheter. For the last three years of his life, he suffered from symptoms resembling those of stone, and, in particular, from pain at the point of the pelvis, bloody urine, &c. Latterly, he complained of tenesmus, and a feeling of stuffing in the rectum, and difficulty in emptying that bowel, as if from the presence of a foreign body in its neighbourhood. He also passed ammoniacal mucus by the urethra. The examination of the prostate by the rectum, nevertheless, detected no enlargement of that organ; and a stone could not be detected by the sound. All the usual expedients were adopted for the relief, both of the vesical and prostatic symptoms, but without the smallest benefit. He died exhausted, after passing from his bladder what appeared to be purulent matter.

An examination of the body revealed the cause of the symptoms. The urethra was perfectly healthy. The prostate was not enlarged. The bladder appeared thin, as if from distention. Between the fundus of the bladder and the umbilicus, in front of the peritoneum, behind the abdominal muscles, and apparently in the substance of the urachus, was situated an abscess of a conical form. The apex of the abscess extended to within an inch of the umbilicus; its base was rounded, and was attached to the fundus of the bladder, but separated from it by a neck or constriction of the peritoneum, presenting the appearance, when viewed from the abdominal aspect, of a second bladder, connected by an isthmus to the fundus of the first. The walls of the abscess were rough and floculated, and its cavity communicated with the bladder by an ulcerated opening, apparently of recent origin.—*Lon. Mon. Jour. of Med. Science.*

